# Photosensitizing Antivirals

**DOI:** 10.3390/molecules26133971

**Published:** 2021-06-29

**Authors:** Kseniya A. Mariewskaya, Anton P. Tyurin, Alexey A. Chistov, Vladimir A. Korshun, Vera A. Alferova, Alexey V. Ustinov

**Affiliations:** 1 Shemyakin-Ovchinnikov Institute of Bioorganic Chemistry, Miklukho-Maklaya 16/10, 117997 Moscow, Russia; mariewskaya.k@gmail.com (K.A.M.); ap2rin@gmail.com (A.P.T.); dobr14@yandex.ru (A.A.C.); v-korshun@yandex.ru (V.A.K.); 2 Higher Chemical College of the Russian Academy of Sciences, Mendeleev University of Chemical Technology, Miusskaya sq. 9, 125047 Moscow, Russia; 3 Gause Institute of New Antibiotics, B. Pirogovskaya 11, 119021 Moscow, Russia

**Keywords:** broad-spectrum antivirals, photosensitization, lipid bilayer, singlet oxygen, hypericin, perylene derivatives, BODIPY dyes

## Abstract

Antiviral action of various photosensitizers is already summarized in several comprehensive reviews, and various mechanisms have been proposed for it. However, a critical consideration of the matter of the area is complicated, since the exact mechanisms are very difficult to explore and clarify, and most publications are of an empirical and “phenomenological” nature, reporting a dependence of the antiviral action on illumination, or a correlation of activity with the photophysical properties of the substances. Of particular interest is substance-assisted photogeneration of highly reactive singlet oxygen (^1^O_2_). The damaging action of ^1^O_2_ on the lipids of the viral envelope can probably lead to a loss of the ability of the lipid bilayer of enveloped viruses to fuse with the lipid membrane of the host cell. Thus, lipid bilayer-affine ^1^O_2_ photosensitizers have prospects as broad-spectrum antivirals against enveloped viruses. In this short review, we want to point out the main types of antiviral photosensitizers with potential affinity to the lipid bilayer and summarize the data on new compounds over the past three years. Further understanding of the data in the field will spur a targeted search for substances with antiviral activity against enveloped viruses among photosensitizers able to bind to the lipid membranes.

## 1. Introduction

Outbreaks of diseases caused by coronaviruses SARS-CoV (2002–2004), MERS-CoV (2012, 2014, 2015), filovirus Ebola (2014–2015, 2018–2019), as well as the current pandemic caused by coronavirus SARS-CoV-2 (2019–to date), has stimulated extensive research on antiviral compounds [1,2,3,4,5,6] and has shown the importance of the availability of approved broad-spectrum antiviral drugs suitable for rapid repurposing or repositioning [7,8,9,10,11,12] to combat emerging threats. The need for such drugs was postulated in 2015 [13,14]. The importance of the problem pushed the search for active antivirals among nanomaterials [15,16,17,18,19,20] and photosensitizers [21,22,23,24,25]. Although photosensitizing antivirals are, in principle, suitable for disinfection purposes (e.g., rooms and vehicles), in this account we are focusing on inhibition of viral reproduction in host cells.

Many dangerous and widespread viruses are enveloped, for example, respiratory viruses, viruses of hemorrhagic fevers and encephalitis, herpes, hepatitis, HIV, etc. Enveloped viruses possess a supercapsid based on a lipid bilayer, which they acquire from membranes of the host cell upon maturation and egress. Origination of the lipids from the host cell provides for considerable similarity of the lipid part of envelope of various viruses (although the exact lipid composition may differ significantly [26]). The lipid bilayer of the virion is as an excellent target for broad-spectrum antivirals [27,28]. Since oxidation of unsaturated lipids by singlet oxygen is well known [29,30], it is assumed that lipid oxidation in the virion envelope disrupts its ability to fuse with the lipid membrane of the host cell, thus preventing the penetration of the viral genetic material inside [28]. Lipid-directed antiviral drugs could be attractive because there is no obvious mechanism for the emergence of virus resistance by mutations, since lipid biosynthesis is not encoded in the viral genome [28]. Unlike the virion, the cell is capable of synthesizing lipids and has repair systems that restore the function of the membrane after oxidative damage to its components, which provides greater cell resistance to oxidants.

Photosensitizers are substances that can exhibit biological activity under electromagnetic irradiation (UV, visible, IR). Upon absorption of a light quantum, the photosensitizer forms the singlet excited state (S_1_) and is then capable of either reacting with biomolecules itself, or, as a result of intersystem crossing (ISC), transform into a triplet state (T_1_). Inactivation of a triplet excited state is possible through the transfer of an electron to a substrate (type I photoreaction) or through energy exchange with a triplet oxygen molecule (^3^O_2_), resulting in formation of singlet oxygen (type II photoreaction) [31,32,33]. Type I photoreaction yields intermediate formation of ion-radicals, subsequently forming various reactive oxygen species (ROS). Either way, ^1^O_2_ or other generated ROS can cause damage to biomolecules [29].

There are many reports of photosensitized antiviral action of various compounds [31,34,35,36,37,38,39]. However, investigation of the mechanism of action and identification of targets for such compounds is a complicated task due to the multidisciplinarity of the problem itself. Often, only observations of an increase of the antiviral effect under light exposure are reported. However, for an unambiguous clarification of the mechanism, a cooperation of photophysics, photochemistry, molecular biology and virology is necessary. All hypotheses about photo-damage to certain molecules (membrane lipids, proteins or nucleic acids), as a result of which certain stages of viral replication are inhibited, are still awaiting careful experimental proof. The presence of a dual (alternative) mechanism of antiviral action for a single compound may an important problem. In addition, photosensitization has a damaging effect on cells, which should be taken into account upon the interpretation of experimental results.

Data on antiviral photosensitizers are summarized in several, including some recent, reviews [31,34,35,36,37,38,39]. The most prominent is a comprehensive review [31] containing more than 600 references on photo-dependent antiviral action. However, it is possible that, on the one hand, for some substances, their ability to photogenerate singlet oxygen or induce oxidative stress under electromagnetic irradiation may be known, and on the other hand, in some reports, the activity of these substances against enveloped viruses can be described without any study of its photo-dependence. Despite the fact that direct evidence of a causal relationship between antiviral activity and generation of singlet oxygen is relatively rare, correlations between these types of bioactivity suggest that this approach is promising for the development of broad-spectrum drugs.

Apparently, the possibilities of photodynamic therapy are limited due to the need for irradiation. However, external viral infections and respiratory viral diseases of the upper respiratory tract treatable by this type of therapy also pose a serious threat. Moreover, rapidly progressing medical techniques allow us to expect the development of methods of exposure to electromagnetic radiation for invasive viral infections (via both instrumental and biophotonic approaches) in the future. Therefore, the identification of patterns in the antiviral activity of photosensitizers for certain types of compounds can spur the development of valuable drugs.

**Figure 1:**
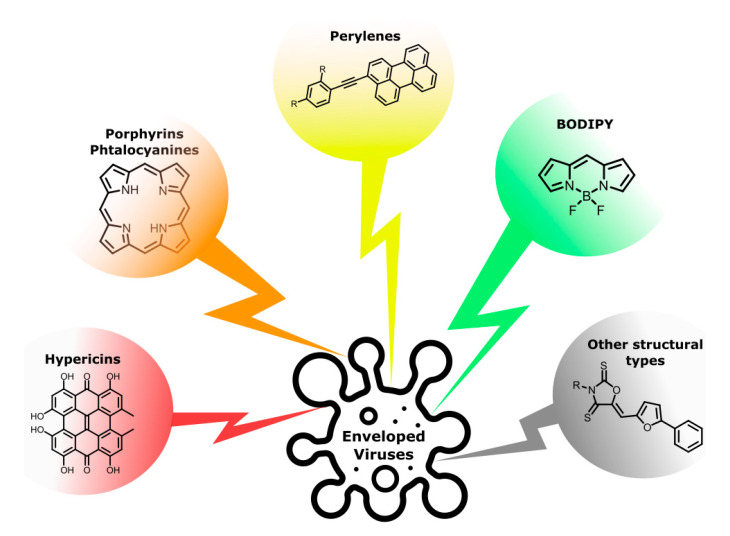
Structural types of promising antiviral photosensitizers with a possible affinity to lipids.

Thus, in this review, we set out to (1) recall the main photoactive antiviral scaffolds with a possible affinity to the lipid bilayer; (2) give data on the antiviral activity of photoactive compounds; and (3) consider the data that have appeared since the last comprehensive account [31]. The review contains sections devoted to some structural types of photosensitizers with antiviral activity (Figure 1).

## 2. Hypericin and Related Compounds

**Figure 2:**
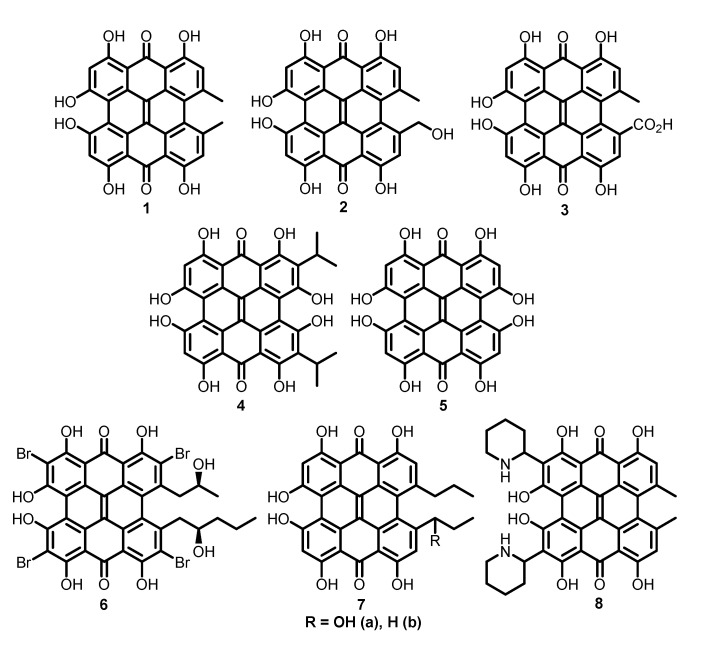
Natural naphtodianthrones.

Hypericin (**1**) and its congener pseudohypericin (**2**) are pigments found in the flowers, leaves and stems of Saint John’s wort, *Hypericum perforatum* (Guttiferae), and most species of the same genus [40]. **1** can also be synthesized by endophytic fungus *Thielavia subthermophila* INFU/Hp/KF/34B [41,42] and mushrooms of subgenus *Dermocybe* (*Cortinarius*) [43]. These polyketides contain condensed naphtodianthrone (or phenanthroperylene quinone) scaffold, which is extremely rare in natural products [44]. Hypericin and related monocarboxylic acid (**3**) have also been found in insects [45]. Similar octahydroxylated compounds have been described for protozoa (stentorin, **4**, from *Stentor coeruleus*) [46,47] and fossil sea lily (fringelite D, **5**, from *Apiocrinus* sp.) [48,49]. Living sea lilies *Gymnocrinus richeri* [50] and *Holopus rangii* [51] produce a series of brominated naphtodianthrones—gymnochromes (gymnochrome A, **6**). Nonbrominated analogs of gymnochromes have also been found in crinoids *Lamprometra palmata gyges* (**7a**) and *Himerometra robustipinna* (**7b**) [52]. Corresponding pigments with secondary amino groups have been identified in the buckwheat flowers (fagopyrin F, **8**, from *Fagopyrum esculentum*) [53,54] (Figure 2).

Hypericin has been intensively studied as the active compound of Saint John’s wort—a well-known traditional medicine. Due to monoamine oxidase inhibition activity of hypericin [55], the extract of *Hypericum perforatum* is used as an antidepressant drug. On the other hand, the fact that **1** has photosensitizing properties and could cause phototoxic reactions was discovered about a century ago [44].

Antiviral activity of hypericins (**1**,**2**) were described in 1988 for the first time by Meruelo et al. [56] on Friend leukemia virus (FV) and radiation leukemia virus (RadLV). In the next few years, activities of **1** against human immunodeficiency virus type 1 (HIV-1) [57], Moloney murine leukemia virus (Mo-MuLV) [58], equine infectious anemia virus (EIAV) [59], vesicular stomatitis virus (VSV), herpes simplex virus (HSV) types 1 and 2, parainfluenza virus, vaccinia virus [60], murine cytomegalovirus (MCMV), and Sindbis virus [61] were reported. Through extensive testing, two important patterns of hypericin’s action have been identified: first, the activity was manifested (or significantly increased) under the influence of light [59,61,62]; second, it was active against enveloped viruses [58]. There is a number of studies utilizing the broad spectrum of hypericin’s antiviral activity for the development of potent agents for virus inactivation [31], for example, against a novel duck reovirus [63].

It was found that hypericin inactivates the viral fusion function by singlet oxygen produced upon illumination [64]. Photoinactivation of viral fusion was observed with hypericin concentrations of 20–50 nm (for VSV) [64]. It is worth noting that hypericin itself is rather inert to singlet oxygen [44]. In some cases, activity was registered in the absence of light and at low levels of oxygen [65]. It indicates that antiviral pathways independent of oxygen photoactivation may exist, but are not predominant. However, a significant reduction of light-induced antiviral activity of hypericin under hypoxic conditions was reported [65]. To illustrate the difficulties in photodynamic studies of antivirals, we would like to give an extensive quotation of a peculiar statement from authors of the paper [65]: “*We had previously reported that hypericin does not require oxygen for its antiviral activity … In those studies, however, we were not able to estimate accurately low oxygen levels in our virus samples. In the present study, we reexamine the importance of oxygen using experimental conditions where the effect of oxygen depletion could be quantified. The results indicate that while antiviral pathways independent of oxygen may exist, the role of oxygen in this activity is significant*”.

**Figure 3:**
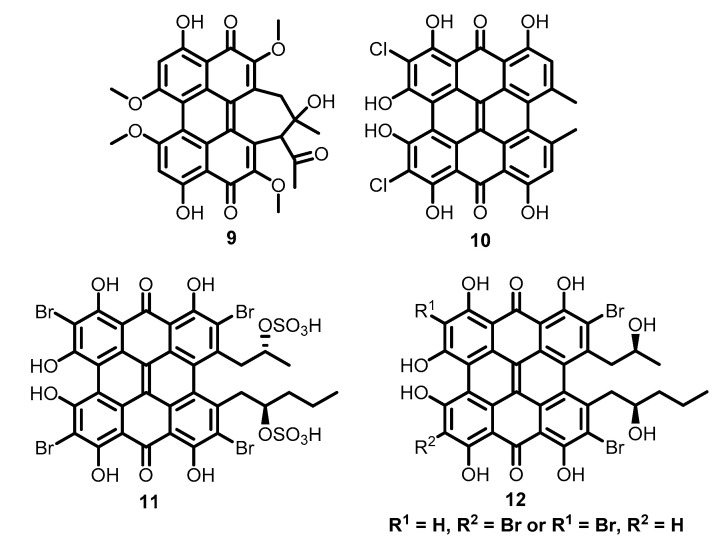
Other antiviral perylene quinone natural products.

Other hypericin analogues also exhibit similar antiviral properties. Natural perylene quinone hypocrellin A (**9**), isolated from fungus *Hypocrella bambusae* [66], is phototoxic to HIV-1 [67], HSV-1, Sindbis virus [68], and VSV [69]. 7,7′-Dichlorohypericin (**10**), isolated from lichens *Nephroma Iaevigatum* [70] and *Heterodermia obscurata* [71], exhibited strong inhibitory activity against HSV-1 [72]. Sulphated gymnochromes—gymnochrome D (**11**) and its atropisomer—are highly potent dengue antiviral agents [73]. Gymnochrome B (**12**) is active against HSV-1, influenza virus, type A [74], dengue viruses, Japanese encephalitis virus [75] (Figure 3). In the last case, ED_50_ activity level was reported as 29 nM with light and 560 nM without light [75].

Thus, hypericin and congeners are capable of photogenerating singlet oxygen. However, do they penetrate the lipid membranes of cells and enveloped viruses? There is no unambiguous answer to this question, although the interaction of hypericin with lipid membranes has been extensively studied [76,77,78]. Convincing evidence in favor of such a mechanism of antiviral activity of hypericin and its structural analogs is the lack of antiviral activity against non-enveloped viruses and an obvious connection between antiviral properties and the generation of singlet oxygen [38].

## 3. Porphyrins, Phtalocyanins and Related Compounds

**Figure 4:**
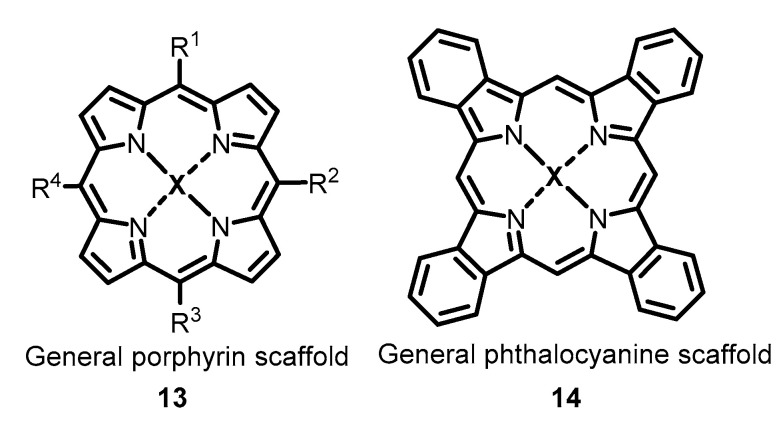
General scaffolds of porphyrins (**13**) and phthalocyanines (**14**).

Porphyrins and porphyrinoids are tetrapyrrole compounds (**13**,**14**) (Figure 4), some of which occur naturally in the human body. The valuable photophysical characteristics of these compounds have attracted considerable interest from researchers due to their use as photosensitizers. Indeed, many drugs used for antitumor photodynamic therapy are based on a porphyrin core [79]. Recently, these compounds have attracted interest for other areas of application, including photodynamic therapy (PDT) of skin infections, including viral [80] and other infectious diseases [81] and in the development of porphyrin nanomaterials in diagnostics and imaging [82]. The properties of porphyrins and phthalocyanines as antiviral sensitizers are mentioned in a review [31]; moreover, data on porphyrin analogs as antiviral agents are summarized in a recent focused account [39]. Most porphyrins act via type II photoinctivation through the generation of singlet oxygen, while the generation of ROS according to type I photoreactions is rather uncommon for these compounds [39].

Most papers on photosensitization are devoted to *meso*-aryl-substituted porphyrins and their metal complexes. Porphyrins are capable of photoinactivation of both enveloped and non-enveloped viruses and damage both lipids and proteins and nucleic acids [83]. Therefore, the structural motifs and structure-activity relationship (SAR) in a series of these compounds differ significantly depending on the specific model and target [39]. It has recently been shown that, depending on the concentration of the photosensitizer, its mechanism of action can change: irradiation with a relatively low concentration of the photosensitizer (octacationic octakis (cholinyl) zinc phthalocyanine) inactivated viral particles, but did not destroy them. Transmission electron microscopy (TEM) revealed that virion membranes kept their structural integrity but lost their surface glycoproteins [84].

Porphyrins and related compounds continue to attract the attention of researchers as a source of viral photoinactivation agents. Recently, an in vitro photoinhibitory effect of Radachlorin in combination with methylene blue against SARS-CoV-2 has been reported [85]. Moreover, water-soluble tetra-cationic porphyrins were found to display light-dependent virucidal activity against Bovine adenovirus (non-enveloped) and Bovine alphaherpesvirus 1 (enveloped) at rather high concentrations, 1.0–5.0 μM, thus illustrating the less selective photodynamic action of porphyrin derivatives [86].

It should be noted that the antiviral action of porphyrins and related compounds can be mediated by a variety of mechanisms, in addition to photoinactivation. For example, cationic *meso*-arylporphyrins, which were previously widely studied as photoinactivators, are also capable of exhibiting antiviral activity in the dark [87]. Recently, the attention of researchers has been attracted by such applications of porphyrinsas inhibition of fusion [88] and binding to G-quadruplex [89]. Nevertheless, even in the case of an unclear mechanism of action, porphyrin-like compounds are still prospective for drug development, because they often have low toxicity levels. For example, a series of synthetic nitrocorroles were found to be excellent candidates for human cytomegalovirus hCMV inactivation (at concentrations as low as 220 nM), exhibiting low toxicity and high therapeutic indices (up to 200) [90].

Thus, porphyrins are exhibiting a wide range of viral inactivation mechanisms, which, on the one hand, makes them promising compounds for the development of disinfectants and drugs, and, on the other hand, complicates rational design of bioactive derivatives, since it often does not allow determining how exactly the observed activity is achieved.

## 4. Perylene-based Rigid Amphipathic Photosensitizers

**Figure 5:**
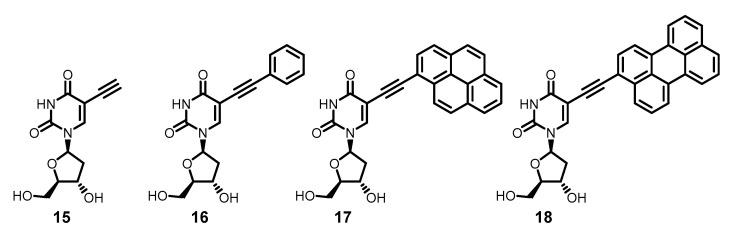
5-Alkynyl-2′-deoxyuridines.

The so-called nucleoside mechanism of antiviral action consists of sequential phosphorylation of nucleoside analogs at the 5′-hydroxyl by intracellular kinases into mono→di→triphosphates, followed by inhibition of DNA polymerases. Increasing the size of the substituent at the 5-position of pyrimidine nucleosides impairs their substrate properties with respect to kinases. Indeed, while 5-ethynyl-2′-deoxyuridine **15** shows antiviral properties against HSV, 5-phenylethynyl-2′-deoxyuridine **16** is already completely inactive [91]. Of course, increasing the size of the aromatic substituent from phenyl (in compound **15**) to tetracyclic pyrenyl or pentacyclic perylenyl (compounds **17** and **18**, respectively) should make phosphorylation even more difficult (Figure 5). Therefore, the discovery of pronounced anti-HSV activity for compounds **17** and **18** might seem surprising [92]. However, everything falls into place if we assume an alternative, non-nucleoside mechanism of action for compounds **17** and **18**.

**Figure 6:**
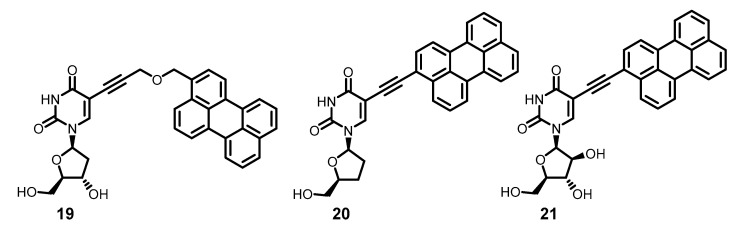
Perylene-based RAFIs.

It was later found that if the rigidity of the molecule in nucleoside derivative **18** is violated by inserting a flexibility element between the ethynyl group and the aromatic residue, the anti-HSV activity of the resulting substance **19** is dramatically reduced [93]. Then it turned out that compounds **18**, **20** and **21** (Figure 6) possess the highest antiviral activity (IC_50_ 5–130 nM, selectivity indices > 3000) compared to their analogues, i.e., those in which a) a perylene residue is present; b) it is linked to uracil by a rigid ethynyl linker, with activity to several envelope viruses—HSV, VSV, HCV, SIN [94], mCMV, IVA [95]—being observed.

This allowed us to hypothesize that the target for such compounds is the virion lipid membrane, which is the common structural element for enveloped viruses, and the mechanism consists of a mechanical incorporation of a hydrophobic perylene fragment into the virion lipid bilayer, which disrupts the rheology of the virion membrane and makes its fusion with the host cell membrane dramatically harder [94]. Therefore, such compounds were named rigid amphipathic fusion inhibitors (RAFIs).

However, a different group of researchers later confirmed the high antiviral activity of compound **18** against HSV-1, VSV, as well as Newcastle disease virus (NDV), Sendai virus (SeV); its antiviral effect was found to be light-dependent, and efficient photogeneration of singlet oxygen by **18** was demonstrated [96]. When HSV-1 virions were preincubated with various concentrations of **18** (**dUY11**) for 30 min in the absence of light and then exposed to a white-light source for an additional 10 min and applied to cells, IC_50_ 0.2 nM was observed. In the presence of sodium azide as a singlet oxygen quencher, the antiviral effect was reduced. Unsaturated virion membrane lipids are postulated to be the target, and the mechanism is considered to be similar to the antiviral action of Broad-SAVE compounds (LJ001 and others, see Section 6).

Since the perylenethynyl chromophore is the same in compounds **18**, **20**, and **21**, one can assume that they all are capable of photoproducing singlet oxygen. It is interesting to note that in typical virological experiments, only mixing of the components—cells, virions, and the antiviral compound—occurs in the light (which usually takes less than an hour), and then virus replication takes place in the dark. It turns out that even a brief exposure of virions to singlet oxygen is enough to damage their lipids and strongly inhibit their fusion with the cell membrane. However, some contribution of a non-photophysical mechanism to the activity cannot be ruled out.

**Figure 7:**
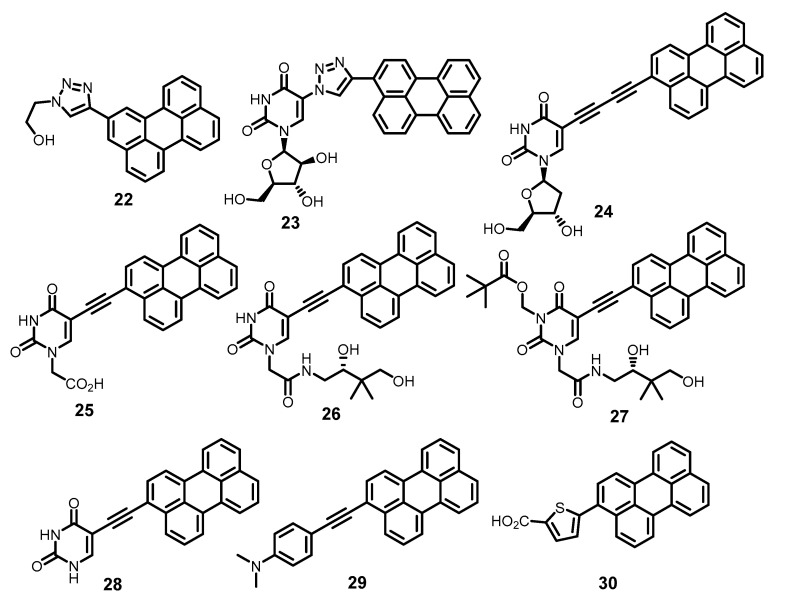
Other antiviral perylene derivatives.

Compounds **18** and **21** showed high activity (EC_50_ 20–25 nM) against tick-borne encephalitis virus (TBEV) [97]. Subsequently, numerous perylene derivatives (e.g., **22**–**30**) (Figure 7) were synthesized [98,99,100,101], showing high activity against TBEV (EC_50_ up to < 1 nM) and HSV, as well as African swine fever virus [102] and respiratory viruses [103]. The structural diversity of antiviral perylene compounds and their action exclusively against enveloped viruses with little cytotoxicity is obvious evidence in favor of the fact that the targets of such substances are the lipids of the virion envelope.

The ability of perylene compounds to photogenerate singlet oxygen is well known. Both perylene itself [104], and perylene-3,4,9,10-tetracarboxylic acid diimides, are efficient in this process [105,106]. The BODIPY-perylene dyad is an effective photosensitizer of ^1^O_2_ formation [107]. Therefore, it can be assumed that photogeneration of singlet oxygen should also be characteristic for perylene derivatives like **22**–**30**.

## 5. BODIPY Compounds

**Figure 8:**
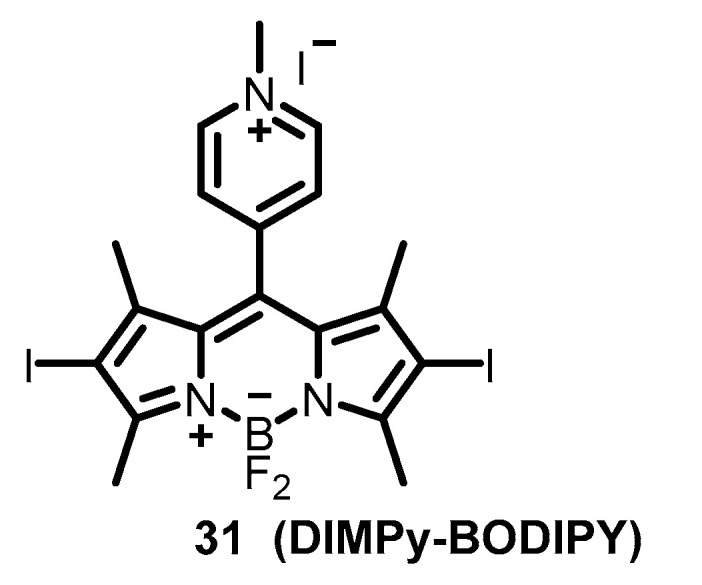
The structure of antiviral BODIPY derivative [111].

BODIPY dyes are widely used in bioimaging due to their outstanding photophysical properties, so their possible application in photodynamic therapy is of considerable interest to researchers [108,109,110]. However, photoinactivation of the virus has been described for only one BODIPY derivative **31** (Figure 8) [111]. Recently, a self-disinfecting material on the basis of this compound showed a complete inactivation of model vesicular stomatitis virus (VSV) [112].

In our opinion, the BODIPY scaffold has great potential as a source of antiviral agents, since its photophysical properties and the ability to generate singlet oxygen have been widely studied, including in the context of photodynamic therapy of microorganisms [107,113,114,115,116]. The applicability of this structural class for virus photoinactivation has already been demonstrated, and the extensive opportunities in fine tuning the photophysical properties of BODIPY derivatives provides a solid foundation for the design of new molecules with valuable properties.

## 6. Other Structural Types

The other structural type of compounds is discussed in detail in previous literature reviews [31]. Here we mention the most prominent types of compounds, prospective for further investigation as antiviral agents.

**Figure 9:**
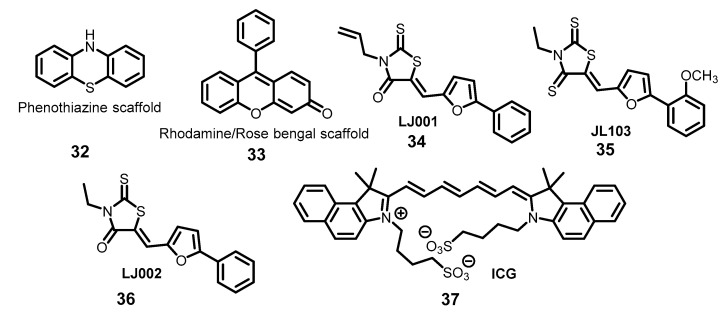
Other aromatic antiviral photosensitizers.

There were some advances in the field of well-known photosensitizers, fused aromatic dyes based on phenothiazine (**32**) and rhodamine (**33**) scaffolds (Figure 9). One of the most widely used phenothiazine photosensitizers is methylene blue, used for blood product disinfection. Recently, this treatment was applied to inactivation of SARS-CoV-2 [85,117]. Production of biocidal reactive oxygen species by rose Bengal was applied for self-disinfecting fabric development in offensive personal protection [118].

The aryl methyldiene rhodanine derivatives (**34**,**35**) (Figure 9) were described as broad-spectrum antivirals [31,119,120,121,122]. Studies of this class of compound have recently advanced significantly, as extensive in vitro and in vivo studies of the mechanism of action for a representative of this family, LJ002 (**36**), have been conducted, showing its high efficacy and low toxicity, which may be a significant advance in the development of drugs based on compounds capable of generating ^1^O_2_ [123].

Another promising scaffold for viral photoinactivation was recently proposed. SARS-CoV-2-RBD was selected as a novel target for indocyanine green (ICG, **37**) (Figure 9) as a photosensitizer in PDT to exploit its molecular modeling, the hierarchical nature of protein structure, and physico-chemical properties using several bioinformatics tools. The binding mode of the RBD to ICG was assessed via protein-ligand docking [124]. Indocyanines are attractive molecules for drug design due to their low toxicity and tunable photophysical properties.

## 7. Conclusions

Although photosensitized antiviral action is known for many classes of substances, a detailed study of the mechanisms and targets seems rather difficult due to the multidisciplinary nature of the area (photochemistry, photophysics, virology) and the need to control many factors (light intensity and time, oxygen diffusion, wavelength of photosensitizer absorption, etc.) Additional difficulties for data comparison arise from the use of different methods for measuring antiviral activity for various viral strains and cell lines. At present, the aggregate of data scattered in numerous articles suggests that the most interesting and promising for the development of broad-spectrum drugs is the type II mechanism—photogeneration of singlet oxygen followed by oxidation of unsaturated lipids of the virion membrane. The mechanism may not be the only one, since some membrane-active substances not absorbing visible/cnear UV light also exhibit antiviral activity. The virion lipid membrane as the target of fusion inhibitor drugs requires the development of new sensitive methods for lipid analysis, as well as methods for computer modeling of drug–lipid membrane interaction. New ideas for the delivery of drugs and illumination to sites of viral replication in the body may also prove fruitful for the development of effective antiviral therapies. The ongoing threat of viral disease epidemics should stimulate research in this challenging area.
